# Racial disparity in the genomics of precision oncology of prostate cancer

**DOI:** 10.1002/cnr2.1867

**Published:** 2023-08-10

**Authors:** Tu Le, Pilar Soto Rojas, Mary Fakunle, Franklin W. Huang

**Affiliations:** ^1^ Division of Hematology and Oncology, Department of Medicine University of California San Francisco California USA; ^2^ Division of Hematology and Oncology, Department of Medicine San Francisco Veterans Affairs Medical Center San Francisco California USA; ^3^ Department of Oncology Hospital Universitario Virgen Macarena Seville Spain; ^4^ Department of Urology University of California San Francisco California USA; ^5^ Chan Zuckerberg Biohub San Francisco California USA; ^6^ Institute for Human Genetics University of California San Francisco San Francisco California USA; ^7^ Bakar Computational Health Sciences Institute University of California San Francisco San Francisco California USA; ^8^ Benioff Initiative for Prostate Cancer Research University of California San Francisco San Francisco California USA

**Keywords:** clinical trial enrollment, Decipher test, germline and somatic test, polygenic risk scores, prostate cancer, racial disparity

## Abstract

**Background:**

Significant racial disparities in prostate cancer incidence and mortality have been reported between African American Men (AAM), who are at increased risk for prostate cancer, and European American Men (EAM). In most of the studies carried out on prostate cancer, this population is underrepresented. With the advancement of genome‐wide association studies, several genetic predictor models of prostate cancer risk have been elaborated, as well as numerous studies that identify both germline and somatic mutations with clinical utility.

**Recent Findings:**

Despite significant advances, the AAM population continues to be underrepresented in genomic studies, which can limit generalizability and potentially widen disparities. Here we outline racial disparities in currently available genomic applications that are used to estimate the risk of individuals developing prostate cancer and to identify personalized oncology treatment strategies. While the incidence and mortality of prostate cancer are different between AAM and EAM, samples from AAM remain to be unrepresented in different studies.

**Conclusion:**

This disparity impacts the available genomic data on prostate cancer. As a result, the disparity can limit the predictive utility of the genomic applications and may lead to the widening of the existing disparities. More studies with substantially higher recruitment and engagement of African American patients are necessary to overcome this disparity.

## 1 INTRODUCTION

Prostate cancer (PCa) is the second most common solid tumor and the fifth leading cause of cancer death in men. In 2020, there were over 1 414 000 estimated new cases of PCa worldwide.^1^ Notably, African American Men (AAM) have the highest risk of developing prostate cancer and also have a higher tendency to be diagnosed at an earlier age compared to European American Men (EAM).^1^ It is well established that significant racial disparities exist regarding PCa incidence and mortality. AAM are 1.8 times more likely to be diagnosed with PCa than men of European ancestry and they also have a 2.4‐fold higher mortality rate.^2^ These differences in the course of the disease progression and survival of patients with PCa are frequently attributed to socioeconomic status and access to medical care, but the cause of the increased PCa risk for AAM is unclear.^3^, ^4^ Even if we adjust for biases attributable to these racial disparities in PCa, incidence and mortality rates remain significantly different among AAM and EAM, suggesting an important contribution of molecular and genetic factors.^5^

In addition to outcome differences between racial/ethnic groups, PCa behaves heterogeneously from patient to patient, making the optimal management strategy a subject of ongoing debate.^6^ The molecular mechanisms of how prostate cancer develops and evolves under therapeutic interventions and the characteristics that make it more aggressive in certain cases remain unclear. To adequately treat these patients, risk stratification models have been created to establish prognosis biomarkers and predict the response to treatments. These models have traditionally been based on clinical and analytical parameters such as stage, Gleason differentiation grade, and prostate‐specific antigen (PSA) value.^7^, ^8^, ^9^ While these features are still useful, their performance in many cases remains suboptimal.^10^ Advances in DNA sequencing and the study of the human genome have made it possible to determine molecular factors that may influence the course of prostate cancer. In the last decade, genome‐wide association studies (GWAS)^11^ have been utilized to translate findings of risk single nucleotide polymorphisms (SNPs) towards clinical utility and to identify genetic predictors of prostate cancer risk. For example, the polygenic risk score (PRS)^12^ is calculated from the sum of the number of risk alleles carried by an individual and weighting each one by its estimated size from GWAS data. This model shows promise in identifying individuals with much higher or lower lifetime risk than the average male and can also improve the predictive value of PSA screening.^13^ For tumor analyses, the Decipher prostate cancer test is a genomic test that is based on the expression of 22 RNA markers and serves as a prognostic marker in patients who have undergone radical prostatectomy (RP). This allows post‐surgical risk stratification and prediction of the probability of metastasis and cancer‐specific mortality to determine the need for adjuvant treatment.^14^ Furthermore, an increasing number of somatic and germline tests are performed in patients with prostate cancer as they determine hereditary risk and guide treatment decisions in cancer.^15^

However, the studies behind these genomic applications, lack racial diversity. In this literature review, we outline the currently available genomic applications to estimate the risk of individuals developing prostate cancer and to identify precision oncology treatment strategies, and how disparities have been approached using these applications.

## 2 POLYGENIC RISK SCORE

Analyses of well‐powered GWAS and next‐generation sequencing (NGS) data have resulted in the identification of numerous SNPs that contribute to overall prostate cancer risk. Although a single SNP itself has a modest predictive power for complex disease outcomes, accumulating various risk‐associated SNPs into a PRS has been shown to improve predictive significance while also providing valuable information for risk stratification.^16^ Prostate cancer, like most cancers, is more likely to be a polygenic disease influenced by a combined effect of multiple genetic variations. To date, GWAS studies have identified 269 common germline genetic variants associated with prostate cancer susceptibility.^17^, ^18^, ^19^ The combined effect of SNPs is estimated to account for a quarter of the familial risk of prostate cancer. Therefore, combining genetic data into a PRS can help predict PCa risk and stratify the probability of developing the disease into high and low risk groups.^20^, ^21^

### 2.1 Clinical utility of PRS

Prostate cancer screening is crucial for attenuating morbidity and mortality.^22^, ^23^, ^24^, ^25^ However, the traditional method of PSA screening is notable for its false‐positive rate and potential harm due to overtreatment^26^, ^27^ The US Preventive Service Task Force recommended the consideration of family history and race/ethnicity to identify men who would benefit the most from early PSA screening. While family history and race/ethnicity are indirect assessments of inherited risk, which can be prone to environmental exposures, PRS creates an opportunity to directly measure inherited PCa risk (Table 1). A cohort study of 3225 men showed that PRS acts as an independent predictor for PCa; thereby, incorporating PRS into the current risk prediction model based on family history, PSA and age could create a better stratification tool with improved predictive capacity.^27^, ^28^ Even among unbiopsied men with low levels of serum PSA of 1–3 ng/mL, PRS can be used to predict biopsy outcomes and identify men with high PCa risk.^29^ PRS's predictive power offers an informative tool to target PSA screening efforts for men with a higher risk of early PCa onset and to guide decisions for a more active clinical approach to those with a higher risk of aggressive PCa.^27^, ^30^ Additionally, there is evidence indicating that the cumulative effect of known polygenic risk factors can modify the risk estimate of mutations in BRCA1 and BRCA2 mutations in male carriers.^31^ PRS can also be applied to other prostate cancer predisposition genes such as CHEK2, ATM, and HOXB13 as a risk modifier to help inform management.^31^, ^32^ This prediction of the independent risk of PCa could help to establish personalized clinical follow‐up recommendations for patients with higher risk and improve early detection and prevention of cancer.

**TABLE 1:** Advantages and disadvantages of prostate cancer screening models.

	Use	Advantages	Disadvantages
PSA levels	Screening	Cheap and universal. We continue to need PSA to predict risk.	High false‐positive rate and potential harm due to overtreatment.
Polygenic risk score (PRS)	Screening	Directly measure inherited PCa risk. Can avoid biopsies when the PSA is doubtful.	Family history and PSA are needed to create a better stratification tool. More expensive and not available in all centers

### 2.2 Disparity in genomic application

Although PRS predictive power is high, PRS studies suffer from a significant deficit in the inclusion of African Americans, which represent only around 3% of the participants for GWASs published through 2015.^33^, ^34^ The insufficient inclusion of African Americans leads to a disproportion in identifying risk‐associated SNPs and compromises the predictive potential of PRS for this minority group.^19^ As a result, these gaps would lead to disparities in how PRS could improve care for patients of European descent compared to patients of African descent.^33^ In a multi‐ethnic study of 80 481 participants, prostate cancer PRS performance was superior in those with genetically defined European ancestry than in those with African ancestry, which comprised 89.3% and 7.8% of the study population, respectively.^28^ This disparity is inevitable considering the bias introduced in European‐dominated GWAS.^28^, ^35^ Furthermore, there is unequal access to PRS between EAM and AAM, which depends on the geographical location of health centers, the health resources available, and the socioeconomic circumstances of the population. Similar to access to other precision approaches in medicine, AAM therefore may not have access to PRS as EAM, and therefore leading to PRS less frequently used in this group. This may limit the predictive power of the test and ultimately cause a delay in the diagnosis and treatment of these patients. On the other hand, the poor inclusion of underrepresented racial/ethnic groups in GWAS studies means that these studies do not provide sufficient data on minorities and we cannot apply them equally to the general population. Such disparities would then lead to inequitable risk stratification.^28^, ^35^

A recent trans‐ancestry GWAS of 127 006 controls and 107 247 prostate cancer cases discovered 86 new risk variants, totaling the known risk variants to 269.^19^ In this study, PRS was shown to have a larger contribution to overall PCa risk for AAM because variants with odds ratio >1.10, which have a greater effect on PRS, are more common in African American participants. Notwithstanding, AAM also have a mean PRS that is approximately 2.18 times higher than that of EAM^19^ and is consistent with the known risk variants that substantially accounted for the estimated 75% higher prostate cancer incidence in African Americans when compared to non‐Hispanic whites.^36^

**FIGURE 1:**
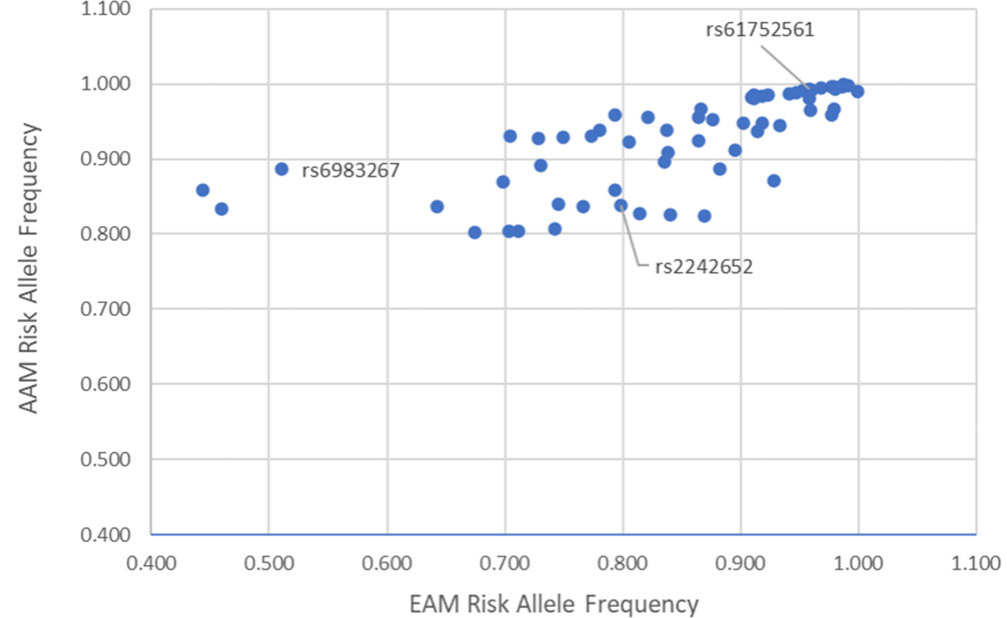
Risk allele frequencies of 61 African American Men (AAM)‐associated single nucleotide polymorphisms (SNPs), European American Men (EAM) versus AAM. SNPs named for the SNPs with potential functional effects are labeled beside their corresponding markers in the scatter plots.

This review filters the 269 known risk variants from Conti's study^19^ to include only the variants highly prevalent in AAM with PCa (those with risk allele frequencies >80% in AAM). A graph of all 269 known risk variants is provided in the Supplementary Material. Figure 1 compares the allele frequencies of these AAM‐associated variants between the AAM and EAM populations and depicts that most of these SNPs are not as prevalent in EAM. Eight of these SNPs, including rs6983267, are located on Chromosome 8, confirming the AAM ancestry associations on 8q24. In AAM, the higher allele frequency within the risk region 8q24 confers a higher incidence of early‐onset PCa and a more aggressive phenotype.^33^ The diagnosis of PCa has also been associated with several additional risk loci at 8q24.^33^ Certain AAM‐associated SNPs may potentially confer functional effects harboring prostate cancer susceptibility: rs2242652 at 5p15 associated with TERT expression which provides a potential biological pathway for increasing PCa risk^37^; and rs61752561on Chromosome 19 has a role for increasing glycosylation, protein stability and PSA activity which suggests potential mechanisms for increasing predisposition to PCa.^38^ However, the samples used to analyze rs2242652 and rs61752561 were predominantly of European ancestry.^37^, ^38^ These results further emphasize the need for AAM inclusion in GWAS studies and the limitation in generalizing the findings across different ancestries.

There has been a considerable effort to attenuate the disparity presented in PRS studies. To compensate for the lack of diversity in GWAS, a study scaled ancestry‐specific PRS distributions that, when considered separately in each ethnic group, can help identify individuals with higher PCa risk in each group.^39^ Another study conducted a cross‐validated search on a dataset that comprised only men with African genetic ancestry and identified three SNPs that significantly improved the performance of PRS in the studied population.^40^ Despite ongoing efforts to enhance diversity within the field of GWAS and improve PRS performance in minority groups, substantial gaps in our understanding persist.

## 3 GENOMICS OF TUMOR BIOLOGY

While PRS measures the risk of developing prostate cancer in the healthy population, Decipher Prostate is a genomic classifier (GC) test for patients with prostate cancer that provides an independent prediction of early clinical metastasis and prostate cancer‐specific mortality following biopsy or RP.^41^, ^42^, ^43^, ^44^ Decipher was created by compiling the genes of 192 metastatic patients with increasing PSA levels over 5 years and comparing them with 271 patients from a retrospective, nested case–control study, culminating in a 22‐gene marker signature, known as the Decipher GC.^45^ Decipher GC incorporates the expression profiles of both coding and non‐coding RNA (ncRNA). Genes involved in metastatic disease progression are significantly affected by ncRNAs. Consequently, other PCa screening tools that overlook the involvement of ncRNA may lose the sensitivity in reporting prognostic information present in GC.^14^ RNA can be extracted from primary prostate cancer specimens that are fixed with formalin and paraffin. After analysis, a Decipher score between 0 and 1 is generated, with low scores of 0.0–0.44, average scores of 0.45–0.59, and high scores of 0.60–1.0. Higher scores indicate an increased likelihood of adverse pathological outcomes while lower GC scores are associated with a lower incidence of metastasis.^42^, ^44^, ^46^ The clinical utility of Decipher has been verified specifically in patients diagnosed with intermediate and advanced PCa.^47^ Further validation is needed to verify the racial disparity in the stratification of PCa progression between AAM and EAM using Decipher GC.

### 3.1 Clinical utility of Decipher

Because Decipher can independently predict metastatic behavior in PCa patients, clinicians can make preliminary treatment decisions by differentiating between low and high‐risk individuals, preferentially treating those predicted with high GC scores.^43^ In comparison, Gleason scores, PSA levels, and other qualitative features of PCa do not adequately distinguish the risk of continued PCa progression and thereby do not offer a proper identification of low and high‐risk PCa patients. By stratifying patients based on their GC scores, personal treatment can be subsequently established. Patients with low GC scores do not necessitate aggressive PCa treatment options such as postoperative radiation, but those with higher GC scores could benefit from aggressive secondary therapy when identified.^47^, ^48^

### 3.2 Disparity in genomic application

The Decipher GC can aid in clinical decision‐making due to its high expected performance. While PRS studies involved screening for PCa predisposition in healthy subjects, most of the studies performed with Decipher are case–control studies in which patients diagnosed with PCa and intervened by RP in a given clinic in the last few years. However, African American patients are underrepresented in most studies evaluating GC, so there is insufficient data on this population.^49^ To include more AAM, studies should focus on centers that serve large minority populations, thereby encompassing a large, diverse, and representative cohort. Several studies have sought to investigate this aspect, recruiting a greater number of African American patients. They demonstrate that the biological characteristics of prostate tumors are different in AAM compared to EAM, and further suggest that African American patients are associated with a higher risk of aggressive disease and have higher decipher scores than non‐African American men.^49^, ^50^, ^51^ The studies also suggest that GC is a stronger predictor in AAM than in EAM.^49^, ^50^ These findings prompt a careful monitoring of African American patients post‐radiotherapy and post‐prostatectomy.

In prostate cancer, various schemes have been developed to classify tumors into subtypes according to the expression of different genes. One study investigated the variation in the distribution and prognostic value of molecularly defined PCa transcriptomic subtype classifiers by race using Decipher.^52^ Five classifiers that identify prostate tumor subtypes were studied and found that the subtypes differed in frequency between AAM and EAM. The classifiers used were: subtypes reflecting luminal or basal lineage; subtypes defined by the presence of ERG fusion, ETS fusion, SPINK1 overexpression; subtypes with early small cell or neuroendocrine differentiation; triple‐negative subtypes, for example, with luminal or basal lineage and ERG or ETS fusions; and Kamoun subtypes such as frequent ERG fusions with inactivation of p53 and PTEN. The association between subtypes and a genomic risk score differed by race suggests that certain subtypes may have a differential prognostic value between racial groups, independent of tumor clinicopathology.^52^ Among AAM, the subtype with luminal lineage, high Gleason score, SPOP mutations, and ETS fusions exhibited a higher frequency. Similarly, AAM displayed a greater prevalence of the triple‐negative, SPINK1 + subtype, and the subtype with an absence of ERG fusions, mutations in SPOP and FOXA1, as well as losses in CHD1 and ZNF292. In contrast, EAM demonstrated a statistically significant prevalence of the subtypes with ERG fusions, inactivation of p53 and PTEN; in addition to the subtypes with ERG fusions and low Gleason score. Certain subtypes frequently found in AAM patients are shown to be associated with higher Decipher scores. However, this association should not be generalized to the general population because the study did not sufficiently include AAM patients.^52^

Another prospective study was conducted to determine the genomic risk of reclassification (GrR) using a clinically balanced cohort of African American men and non‐African American men. GrR is the probability that a tumor, initially classified as low risk or favorable intermediate risk by conventional clinical risk classifiers, ultimately harbors genomic risk of metastasis when assessed by Decipher. This study found that the majority of AAM had a higher Decipher score than EAM, thus AAM was twice as likely to experience a genomic risk of reclassification.^53^ Gleason grade (GG) groups are established based on the architectural features of prostate cancer specimens and help establish a prognosis. The scale ranges from 1 to 5 such that higher GGs indicate a greater likelihood of non‐organ‐confined disease and a poor response to treatment. In a multi‐institutional retrospective analysis of 1152 patients (596 AAM and 556 EAM), Decipher score was compared with GG groups, and a positive relationship was found between these two parameters such that a higher GG is associated with a higher Decipher score.^51^ The study also found that AAM have a higher Decipher score only in the lower GG group (GG1/2). However, the average genomic‐risk score (average of 19 signatures excluding Decipher) is significantly lower in AAM when compared to EAM with the high GG group (GG 4/5) group. These findings imply that racial disparity existed might be more profound in the lowest and highest GG groups.^51^ These differences in the tumor biology between AAM and EAM may provide an explanation for the racial disparities in prostate cancer.

## 4 GERMLINE AND SOMATIC TESTING

The decline in cost and the expansion in the availability of NGS have enabled the application of germline and somatic testing in routine clinical practice.^54^, ^55^, ^56^ While PRS and tests like Decipher help determine the risk of PCa and the risk of recurrence and prognosis from primary prostate cancer, germline and somatic testing determine heritable risk and guide treatment decisions in advanced disease settings.^57^

The assessment of germline genetics helps assess an individual's specific cancer risk, aids family cancer screening, and also informs treatment possibilities.^57^ Somatic mutations are acquired over the course of an individual lifespan. Identification of somatic mutations requires DNA sequencing from tumor tissue, circulating tumor cells, or circulating tumor DNA (ctDNA) in the blood. Since somatic mutations can change over time due to genomic instability and tumor evolution, repeated somatic testing might be appropriate as cancer progresses through treatment.^57^ Identification of germline and somatic mutations can help guide treatment options in the advanced disease setting. Tumor genetic testing can identify both somatic and germline mutations. However, tumor testing should not be performed as a substitution for germline testing given challenges in distinguishing between somatic and germline mutations. In the event that there is an identification of somatic mutation which has implications for cancer predisposition (BRCA1), then a confirmatory germline test is highly recommended.^57^, ^58^

### 4.1 Clinical utility of germline and somatic testing

In the era of precision medicine, genetic testing is widely considered in clinical practice as it helps to tailor the treatment for complex and heterogeneous diseases such as prostate cancer. By sequencing the tumor genome with NGS, actionable biomarkers can be identified.^54^

With approximately 5%–10% of oncogenic mutations being germline mutations,^59^ germline testing is essential in identifying risk biomarkers or germline mutations that are associated with increased cancer susceptibility. In the setting of PCa, the highest risk levels were reported in the presence of homologous recombination repair gene BRCA1/2, which confers a 4‐ to 8‐fold increase in risk,^33^, ^60^ followed by the presence of HOXB13 mutation, a gene encoding homeobox transcription factor B13, which has been associated with a 4 fold increase in susceptibility.^61^, ^62^ Studies have also shown that pathogenic mutations in BRCA1/2 and HOXB13 increase the risk for earlier onset of PCa.^60^ Men with DNA mismatch repair gene mutations (*MLH1*, *MSH2*, and *MSH6*) have a 2‐ to 4‐fold greater susceptibility to develop PCa.^63^ Emerging data suggest that NBS1, FANCA, and other DNA repair genes are associated with increased PCa risk and may inform treatment choices.^64^

The implications of germline and somatic mutations extend to their potential utilization as molecular targets for various pharmaceutical interventions. The TOPARP‐A trial^65^ revealed an improved response to the PARP inhibitor (PARPi), olaparib, among men with metastatic castrate‐resistant prostate cancer (mCRPC) exhibiting DNA‐repair defects (BRCA1/2 and ATM). Following this trial, the United States Food and Drug Administration granted Breakthrough Therapy designation to olaparib. Moreover, the presence of alterations in BRCA1/2 and other DNA repair genes has been correlated with enhanced response to platinum‐based chemotherapy. Similarly, the identifications of DNA mismatch repair genes has been associated with favorable response outcomes in the context of anti‐PD‐1 immunotherapy.^66^, ^67^ With new advances in drug development, more clinically actionable mutations will likely be established and more diagnostic and therapeutic implications for somatic and germline testing will emerge. Continued efforts are needed to determine if these emerging targeted therapies have the same clinical utility in diverse patient populations.

### 4.2 Disparities in genomic application

As genomic data guide subsequent therapy, differential access to genomic testing can widen disparities in clinical trial participation. To date, AAM have been underrepresented in germline and somatic genetic studies of prostate cancer.^67^ The lack of racial diversity in current genetic studies has the potential to exacerbate disparities in health care.

As a result of the deficiency in racial representation in genetic studies, the genetic variants associated with increased cancer risk in AAM and other minority groups are likely to be overlooked. Understanding genetic variants observed across different racial groups might offer insights into the underlying biological factors contributing to the higher prevalence and unfavorable prognosis in AAM with PCa. In a multi‐institutional retrospective analysis, clinicopathological and genomic characteristics were compared between the EAM and AAM racial groups.^51^ European American men were associated with increased ERG and ETS expression and decreased SPINK1 expression. The AAM group was associated with higher expression of CRYBB2, GSTM3 and with increased expression of SPINK1.^51^ In the Asian population, on the other hand, the rates of ERG oncoprotein‐positive prostate cancer are low (13%–22%), whereas SPINK1+ rates are quite similar to that observed in EAM.^68^ In Schumacher et al.^69^ study, it was observed that mutations in ZFHX3 as well as focal deletions in ETV3 were more frequent in tumors from AAM compared to EAM, while ZFHX3 alterations were more common in Asian men compared to both EAM and AAM. MYC amplifications were also more frequent in tumors from AAM men with metastatic PCa than from EAM. TMPRSS2 and FOXA1 alterations were more prevalent in EAM in the metastatic setting^69^ than in African American population; however, FOXA1 was more frequently mutated in Asian than in European populations.^68^ Recent data suggest that AAM with PCa exhibits genetic alterations in highly penetrant germline genes as well as low‐penetrant SNPs.^33^ Higher rates of germline variants of uncertain significance (VUS) have been reported in the African American population compared to individuals of European ancestry with PCa, the meaning of which remains to be elucidated. More studies are needed to facilitate the possible reclassification of VUS.^33^ Studies evaluating germline and somatic alterations in African American men have shown that there is no significant difference in the mutation rate of actionable genes between AAM and EAM with PCa.^67^, ^68^, ^69^, ^70^, ^71^ It is possible that differences in these mutations could reach statistical significance if the population of AAM was augmented, thus allowing firm conclusions to be drawn that hold clinical implications.

**FIGURE 2:**
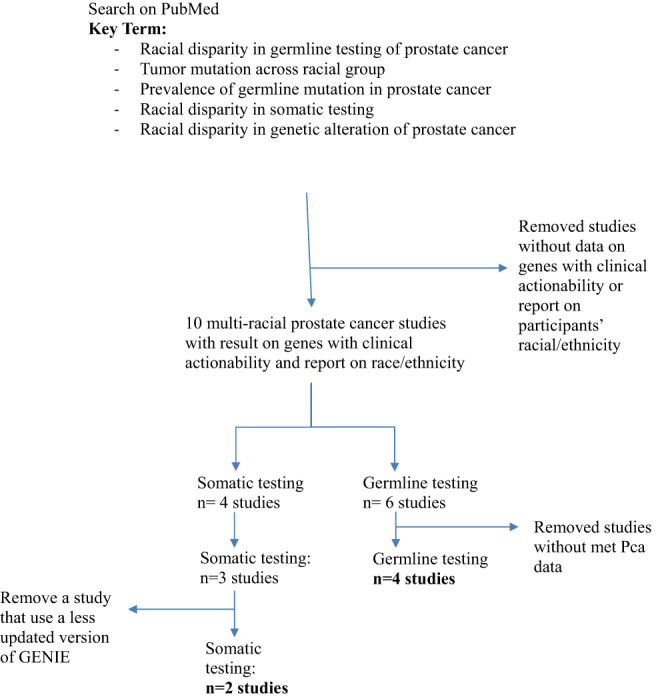
Bibliographic search for studies with actionable mutations and participants.

In the metastatic setting, germline and somatic genetic testing is an integral part of clinical management.^72^ This review will focus on comparing the mutation rate of actionable genes between AAM and EAM with metastatic PCa (the stage of the disease that has a higher mutational burden), as seen in Table 1. In order to achieve this, we conducted a bibliographic search in PubMed to search for multi‐racial prostate cancer studies with results on genes possessing clinical actionability and a report on race/ethnicity. We used the keywords: Racial disparity in germline testing of prostate cancer, tumor mutation across racial groups, prevalence of germline mutation in prostate cancer, racial disparity in somatic testing of prostate cancer, and racial disparity in genetic alteration of prostate cancer. We found four studies analyzing PCa somatic mutations and six studies analyzing PCa germline mutations in the metastatic or lethal PCa setting. We then removed studies that did not have data for metastatic or lethal prostate cancer, which includes the Sartor et al. study, Kwon et al. study, and Nicolosi et al. study. Finally, we did not include the Schumacher et al. and Mahal et al. study because they used previous versions of GENIE included in the Kamran et al. study. In the end, we included two studies of somatic mutations: the Koga et al.^70^ study which includes data from the MC3 (Multi‐Center Mutation Calling in Multiple Cancers) call set from the Pan‐Cancer Atlas Project of The Cancer Genome Atlas (TCGA), from the Foundation Medicine cohort and also from prostate cancers profiled with the Memorial Sloan Kettering‐Integrated Mutation Profiling of Actionable Cancer Targets (MSK‐IMPACT); and the Kamran et al.^72^ study that uses data extracted from the American Association for Cancer Research Project Genomics Evidence Neoplasia Information Exchange (GENIE), version 8.1. In summary, we analyzed a total of 6 studies (2 studies analyzing somatic mutations and 4 studies analyzing germline mutations) (Figure 2). We identified the most frequent actionable mutations in PCa in these studies and analyzed the rates of somatic and germline mutations expressed in the different ethnic groups.

#### 4.2.1 BRCA 1 and 2

BRCA1 and BRCA2 mutations are associated with aggressive PCa with a higher risk for nodal and distant metastasis as well as poor survival outcomes.^73^, ^74^ Two studies identified a similar rate of BRCA2 somatic mutations among EAM and AAM (4.45%–10.02% in EAM and 4.23%–9.73% in AAM with metastatic PCa).^70^, ^73^

In the case of germline variants, three separate multi‐racial studies identified an incidence of BRCA1 germline mutations in 0.5%–0.87% of EAM compared to 0%–2.0% in AAM with metastatic PCa. Four different studies noted a frequency of BRCA2 germline mutations in 3.07%–5.1% of EAM and in 0%–4% of AAM in the context of metastatic PCa (Table 2).^67^, ^71^, ^74^, ^75^ In a study comprised of 2098 AAM and Ugandan men, the largest study of germline mutations in African descendants, BRCA1 mutations were found in 0.72% and BRCA2 mutations were identified in 2.1% of metastatic PCa patients.^75^

**TABLE 2:** Multi‐racial cohorts assessing the prevalence of mutation in genes with established and emerging clinical actionability in AAM and EAM with metastatic PCa, somatic and germline test.

Mutation	Associated with increased cancer risk	Therapeutic indication	Somatic test for metastatic PCa			Germline test for metastatic PCa		
			Mutation rate in European descendant (%)	Mutation rate in African descendant (%)	Reference	Mutation rate in European descendant (%)	Mutation rate in African descendant (%)	References
BRCA 1	x	PARPi				0.5% 0.77% 0.87%	2.0% 0.0% 0.0%	Ledet et al. Na et al. Pritchard et al.
BRCA 2	x	PARPi	4.45% 10.02%	4.23% 9.73%	Kamran et al. Koga et al.	5.0% 3.07% 4.77% 5.10%	4.0% 0.0% 0.0% 0.90%	Ledet et al. Na et al. Pritchard et al. Plym et al.
ATM	x	PARPi	7.29% 5.75%	9.86% 3.78%	Kamran et al. Koga et al.	1.0% 1.53**%** 1.01%	0.0% 3.33% 0.14%	Ledet et al. Na et al. Pritchard et al.
PALB2	x		0.83%	2.16%	Koga et al.	0.50% 0.43%	0.50% 0.0%	Ledet et al. Pritchard et al.
CHECK2	x	PARPi	2.32%	1.08%	Koga et al.	2% 1.01%	0.0% 0.0%	Ledet et al. Pritchard et al.
MSH2	x	Checkpoint inhibitor	1.30%	1.62%	Koga et al.	0.20% 0.14% 0.70%	0.0% 0.0% 1.80%	Ledet et al. Pritchard et al. Plym et al.
MSH6	x	Checkpoint inhibitor	1.76%	1.08%	Koga et al.	0.50% 0.14% 0.30%	0.50% 0.0% 0.00%	Ledet et al. Pritchard et al. Plym et al.
PMS2	x	Checkpoint inhibitor				0.30% 0.70%	0.50% 0.00%	Ledet et al. Plym et al.
FANCA	x	PARPi	1.11%	0.00%	Koga et al.	0.30%	0.00%	Plym et al.
NBN	x	PARPi	0.65%	1.08%	Koga et al.	1% 0.14% 0.00%	0.0% 0.0% 0.90%	Ledet et al. Pritchard et al. Plym et al.
CDK12		Checkpoint inhibitor	5.55% 4.73%	11.27% 7.57%	Kamran et al. Koga et al.			

#### 4.2.2 ATM

ATM mutations have been associated with aggressive PCa.^74^, ^76^, ^77^ Two separate multi‐ethnic studies found no significant difference in ATM somatic mutation rate between EAM and AAM with metastatic prostate cancer (5.75%–7.29% and 3.78%–9.86% respectively). Three separate studies also noted similar rates of ATM germline variants in EAM with metastatic PCa (1%–1.53%) compared to the reported ATM mutation rates among AAM with metastatic PCa (0%–3.33%) (Table 2).^67^, ^71^, ^74^ In a study with more than 2000 men of African ancestry, the incidence of ATM germline variants was 1.8% in patients with metastatic PCa.^77^

#### 4.2.3 PALB2

PALP2 mutation is associated with a 6.3 higher risk for aggressive PCa.^78^ There was no statistically significant difference observed in the rate of PALB2 somatic mutation between EAM (0.83%) and AAM (2.16%) with metastatic PCa (Table 2).^70^ The rates of PALB2 germline mutation were similar in both racial groups with a range spanning from 0.43% to 0.5% in EAM and 0% to 0.5% in AAM with metastatic prostate cancer (Table 2).^67^, ^71^

#### 4.2.4 CHEK2

CHEK2 is a tumor suppressor gene located on chromosome 22q, and its mutations exhibited a significantly higher prevalence in men with metastatic PCa compared to those with localized PCa. CHEK2 encodes a cell cycle checkpoint protein kinase that plays a role in the regulation of p53 (TP53) and DNA repair. The rate of CHEK2 somatic mutations was 2.32% in EAM and 1.08% in AAM with metastatic PCa.^70^ Studies have reported CHEK2 germline mutation in the range of 1.01%–2% in EAM and 0% in AAM with metastatic PCa (Table 2).^67^, ^71^

#### 4.2.5 Lynch syndrome

Lynch syndrome is an inherited cancer predisposition syndrome with an increased risk of numerous malignancies, doubling the risk of PCa. It is caused by germline mutations in the mismatch repair genes MLH1, MSH2, MSH6, and PMS2.^79^ The studies included in the analysis showed somatic mutation rates in the EAM population ranging from 1.30% to 1.76%. Similarly, the AAM population with metastatic PCa presented a comparable expression rate, with a range spanning from 1.08% to 1.62%.^70^ Regarding germline mutations in the metastatic PCa setting, the EAM population presented a range from 0.14% to 0.70%, while the AAM population exhibited a similar range from 0.0% to 1.80% (Table 2).^67^, ^71^, ^75^

#### 4.2.6 NBN

NBN encodes for Nibrin, a protein within the Mre11‐RAD50‐NBS1 double‐stranded DNA break repair complex. NBN has recently been identified as a PCa‐susceptibility gene.^80^ Regarding the NBN mutation in the context of metastatic PCa, the included studies revealed a somatic mutation rate of 0.65% in EAM, whereas the AAM population presented a rate of 1.08%.^70^ The EAM population presented a germline mutation rate of 0.0%–1% while the AAM population displayed an NBN germline mutation rate of 0.0%–0.9% (Table 2). None of these mutation rates exhibited statistical significance in terms of racial differences ^67^, ^71^, ^75^

#### 4.2.7 CDK12

The prevalence of CDK12 somatic mutations in the setting of metastatic PCa is higher in AAM (7.57%–11.27%) than in EAM (4.73%–5.55%) (Table 2),^70^, ^73^ but this difference is not statistically significant. Nevertheless, the study Koga et al.^70^ reported a statistically significant difference in CDK12 deletion, a subtype of CDK12 mutation, with a higher mutational frequency in AAM than in EAM with metastatic PCa (2.16% vs. 0.18%. respectively).

### 4.3 TP53, PTEN and TMPRSS2‐ERG mutations

Analyzing and comparing the percentage of patients in each study, we observed that the Ledet et al.^67^ trial included a total of 867 patients, of which 188 were African American (21.6%) and 669 (77.2%) were White patients. Kamran et al.^73^ evaluated a total of 20 191 patients with various types of tumors, of whom >80% were White patients and only 8.6% were AAM. Na et al.^74^ included a total of 799 patients, of which 613 were EAM (76.7%), 119 patients were AAM (14.9%), and 67 patients (8.4%) were of other races. Out of 764 PCa cases analyzed in the Plym et al.^75^ study, 188 (24.6%) were AAM and 576 (75.4%) were EAM. Lastly, the Koga et al.^70^ study included 861 patients with PCa, of whom 250 men were AAM (29.0%) and 611 men were EAM (71.0%). The studies with the most African American representation in germline studies were Ledet et al.^67^ and Plym et al.^75^ with 21.6% and 24.6% of participants being AAM respectively. As for somtatic studies, the research conducted by Koga et al. demonstrated the highest AAM representation with 29.0% of participants being AAM. Nevertheless, the AAM population continues to be underrepresented in research studies, thereby impeding the generalizability of the findings. A more racially inclusive cohort is needed to produce more representative data.

We examined the specific racial differences between the mutation rate of the top 5 mutations observed in metastatic PCa: AR, TP53, PTEN, TMPRSS2‐ERG, and RB1. Among these 5 mutations, TP53, PTEN, and TMPRSS2‐ERG displayed significant differences in mutation rates between AAM and EAM with metastatic PCa according to more than one study. In light of these findings, we focused our attention on these three mutations to analyze the differences between the two racial groups specifically in metastatic PCa (Table 3). TP53, PTEN mutations, and TMPRSS2‐ERG fusion are among the most prevalent genetic defects found in lethal metastatic prostate cancer. Koga et al.^70^ showed that in prostate cancer, TMPRSS2‐ERG rearrangements (31.5% in EAM vs. 14.1% in AAM) and PTEN deletions (37.5% in EAM vs. 24.3% in AAM) were less frequent in AAM than in their EAM counterparts. AAM had a 43.2% mutation frequency in TP53 mutation, comparing to 45.3% in EAM. An analysis by Kamran et al.^73^ also demonstrated a similar trend: TP53 mutation in 38.1% of EAM participants and in 25.4% of AAM; PTEN aberration in 10.4% of EAM and in 7% of AAM; and TMPRSS2‐ERG fusions in approximately 30% of EAM and 17% of AAM. Even though various studies analyzing these genetic aberrations in the context of metastatic PCa have yielded consistent findings where AAM demonstrated lower mutational frequencies in these genes compared to their EAM counterparts,^67^, ^68^, ^69^, ^70^, ^71^, ^72^, ^73^ further research is needed to substantiate the racial differences in mutational frequencies in these specific genes.

**TABLE 3:** The prevalence of TP53, PTEN, and TMPRSS2‐ERG mutations in AAM and EAM with metastatic PCa.

Mutations in metastatic PCa	Mutation rate in European descendant (%)	Mutation rate in African descendant (%)	Reference
TP53	45.3% 38.1%	43.2% 25.4%	Koga et al^70^ Kamran et al^73^
PTEN	37.5% 10.4%	24.3% 7%	Koga et al^70^ Kamran et al^73^
TMPRSS2‐ERG	31.5% ~30%	14.1% ~17%	Koga et al^70^ Kamran et al^73^

Interestingly, these findings of lower frequencies in known cancer genes raise questions about the observed aggressive features of prostate cancer in AAM. It is hard to imagine how less frequent deletions of these genes can contribute to more aggressive features of prostate cancer in AAM and higher rates of prostate cancer. The lower prevalence of PTEN mutation, TP53 aberration and TMPRSS2‐ERG fusion among AAM tumors suggests that other molecular alterations or pathways are likely to account for racial disparities in PCa outcomes. Therefore, if precision oncology approaches solely targeted these mutations, such strategies might further exacerbate racial disparities in PCa.

### 4.4 Racial disparity in clinical trials enrollment

Despite racial disparities in prostate cancer incidence and outcome, there is a low enrollment of AAM patients in clinical trials. In a study analyzing 72 prostate cancer trials with start dates between 1987 and 2016, EAM accounted for 96% of all trial participants.^81^ With the advancement of precision medicine, it is imperative to improve representation in trial enrollment in order to ensure properly validated biomarkers, and subsequently, an appropriate treatment decision for the general population, especially for those from marginalized and high‐risk backgrounds.^82^ Here we identify clinical trials started after 2016 that investigated personalized therapy for prostate cancer in order to examine the representation of AAM across prostate cancer precision therapy trials. We carried out a literature search on clinical trial registries (ClinicalTrials.gov) using the keywords: PDL‐1, Pembrolizumab, Nivolumab, Cemiplimab, Durvalumab, PARPi, Veliparib, Olaparib, Niraparib, Rucaparib, Talazoparib, Lutetium, and 177Lu. The objective was to find clinical trials that started after 2016, encompassing those that have been completed or remained active but no longer enrolling patients, and featuring results along with reports of participant race/ethnicity. We eliminated trials that did not meet this condition and we found a total of 14 trials. Of these, we eliminated 1 trial that was not relevant, leaving us with 13 trials.^83^, ^84^, ^85^, ^86^, ^87^, ^88^, ^89^, ^90^, ^91^, ^92^, ^93^, ^94^, ^95^

In the last decade, there has been unprecedented progress in treatment options for patients with prostate cancer resulting in an ever‐growing range of options. This advancement raises the question of which therapies are appropriate for individual patients to receive optimal therapy. Unfortunately, despite new advances in the field, the representation of African American men in these clinical trials to determine whether racial/ethnic differences may affect clinical benefit remains insufficient.^96^, ^97^

Examining the trials activated after 2016, we found that the inclusion of African American patients remains low (<5%) compared to that of European American patients in prostate cancer clinical trials for precision oncology, with a notable lack of African American patients in the trial NCT03093428 (Table 4). The future of precision oncology of PCa is based on these ongoing clinical trials, so the insufficient enrollment of African American participants might limit the generalizability of results to this group of patients. Therefore, existing disparities in trial enrollment may have an impact on the potential benefit in survival, quality of life, and optimal therapies for African American patients.

**TABLE 4:** Representation of race/ethnicity in prostate cancer clinical trials using personalized therapy.

Clinical trial identifier	Study name	Study start year	Investigated drug	Prostate cancer disease	No. participants	No. EAM	% EAM	No. AAM	%AAM
NCT02923180^83^	MGA271	2017	Enobilituzumab	Localized Intermediate and High Risk PCa	32	30	93.8%	1	3.1%
NCT02952534^84^	TRITON 2	2017	Rucaparib	mCRPC	277	198	71.5%	16	5.8%
NCT02987543^85^	PROfound	2017	Olaparib	mCRPC	387	248	64.1%	8	2.1%
NCT03016312^86^	IMbassadore250	2017	Atezolizumab	mCRPC	771	577	74.8%	15	2.0%
NCT03093428^87^		2017	Pembrolizumab	mCRPC	42	39	92.9%	0	0.0%
NCT03148795^88^	TALAPRO‐1	2017	Talazoparib	mCRPC	127	110	86.6%	4	3.2%
NCT03179410^89^	PICK‐NEPC	2017	Avelumab		15	11	73.3%	4	26.7%
NCT03204812^90^		2017	Durvalumab and Tremelimumab	mCRPC	26	22	84.6%	2	7.7%
NCT03338790^91^	CheckMate 9KD	2018	Nivolumab	mCRPC	292	248	84.9%	15	5.1%
NCT03406858^92^		2018	Pembrolizumab and HER2Bi‐Armed Activated T Cells	mCRPC	13	12	92.3%	1	7.7%
NCT03511664^93^	VISION	2018	177Lu‐PSMA‐617	mCRPC	831	721	86.8%	55	6.6%
NCT03516812^94^		2018	Olaparib	CRPC	36	25	69.4%	1	2.8%
NCT04089553^95^		2019	AZD4635, Oleclumab and Duravalumab	mCRPC	59	47	79.7%	8	13.6%
				Total	2908	2288	78.7%	130	4.5%

Approximately 12% of men in the United States are African American.^96^ Since AAM have twice the risk of developing PCa compared to EAM, a proposed representation could be 24% in clinical trials. However, the current average enrollment of AAM to prostate cancer clinical trials overall is only around 3%.^96^, ^97^ Only one of the trials that we analyzed (NCT03179410^89^) included a number of AAM patients that exceed the recommended percentage of 24%. However, the remaining trials did not meet this recommendation.

It is necessary to look for solutions to recruit more African American patients in the trials that are being carried out and to identify the factors that influence the lack of inclusion of these patients in the studies. As such, the lack of patient education could be addressed by establishing health promotion plans in health centers. Another possible problem could be the lack of socioeconomic resources for minority groups, which could be improved by increasing the number of health centers in the most marginalized areas, encouraging public transport to bigger clinics when it is necessary, or establishing economic aid plans for the population to improve access to health care.^98^, ^99^, ^100^

## 5 CONCLUSION

A majority of prostate cancer studies present an underrepresentation of minority populations. This comprises a fundamental problem since African American patients have the highest risk of suffering from PCa and the highest risk of tumor aggressiveness.^101^ In addition, African Americans are more likely to be diagnosed with more advanced diseases when treatment options are typically more limited and less effective.

There are known differences in the biological characteristics of prostate tumors between AAM and EAM,^51^ so it is crucial to improve the representation of AAM in prostate cancer studies in order to better elucidate and investigate these biological differences. The current deficit in African American participants in prostate cancer studies poses a potential limitation to the predictive power of genomic applications such as PRS or Decipher, which are utilized to assess risk in PCa. As a result, this underrepresentation may give rise to gaps in the recommendations that can be provided to this population. Available genomic data on prostate cancer are also affected by the underrepresentation of African American men in germline and somatic genetic studies of the disease.^70^ The inadequate racial inclusion may hinder the ability to translate findings to clinical care and subsequently, the ability to offer personalized treatment. These limitations might exacerbate the existing racial disparities in prostate cancer outcomes.

The underlying reasons for these disparities are multifactorial, necessitating comprehensive efforts to address them at all levels. Health disparities are often attributed to the lack of socioeconomic resources for minorities, leading to reduced accessibility to healthcare.^96^, ^102^ Additionally, the difficulties in completing visits for clinical trials may also arise due to the geographical distance to cancer centers and a lack of transportation support. Improving accessibility to studies is an important factor to take into account, perhaps achieved by increasing fundining to support the treatment, housing, and transportation for underrepresented minorities enrolled in a study.

We must identify and address the differences that occur throughout the diagnostic and therapeutic process in prostate cancer between EAM and racial minorities. Structural, social, environmental, biological, and health factors may influence racial differences in disease outcomes. To further exacerbate the racial disparities, inequality can often be found in the screening processes, in the appropriate use of definitive treatment, in the access to systemic therapies, and in the subsequent follow‐up process. Few studies have defined and addressed these disparities.^98^, ^99^, ^100^ Possible solutions could involve establishing a free population‐wide screening using PSA analysis, equitably allocating resources to all hospitals to ensure universal patient access, or developing new policy strategies to mitigate disparities in access to health care. Further research would be needed to identify potentially addressable causes in order to ensure equality in healthcare.

Another potential barrier lies in the lack of access to information about the studies and trials. Therefore, efforts should be directed toward improving the understanding of the demographic makeup of institutional catchment areas and increasing community outreach and engagement to promote greater diversity in study participation. It is also necessary to establish national support and dissemination programs for the ongoing trials in each center and substantially increase attention to the recruitment capacity of centers. By improving the ability to share information to the community, the recruitment of African American patients in different studies can be enhanced, thus yielding representative and high‐quality data. Engaging in educational initiatives, such as classes or courses in institutes, health centers and in the most marginalized neighborhoods, may be a promising approach in this regard. Finally, addressing longstanding mistrust between the African American population and the health care system is crucial, stemming from past mistreatment of African Americans in research studies such as the Tuskegee Airmen Syphilis Study^103^ and the Henrietta Lacks case.^104^ Improving the racial representation of healthcare providers may help addressing this mistrust since patients from minority backgrounds are reported to be more likely to enroll in research studies when they are approached by providers from similar racial backgrounds.^104^, ^105^ In summary, substantial efforts should be dedicated to improving the diversity of patients included in genomic and clinical studies to generate more evidence that will ultimately inform the most effective and personalized treatments. 

## AUTHOR CONTRIBUTIONS

**Tu Le:** Conceptualization (lead); data curation (lead); formal analysis (lead); resources (lead); visualization (lead); writing – original draft (lead); writing – review and editing (lead). **Pilar Soto Rojas:** Conceptualization (lead); data curation (lead); formal analysis (lead); resources (lead); visualization (lead); writing – original draft (lead); writing – review and editing (lead). **Mary Fakunle:** Writing – review and editing (equal). **Franklin W. Huang:** Supervision (lead); writing – review and editing (equal).

## CONFLICT OF INTEREST STATEMENT

The authors declare no conflicts of interest.

## Supporting information


**Supplemental Figure 1.** Risk allele frequencies of 269 PCa SNPs, EAM versus AAM. The SNPs data were collected from Conti et al. study.^19^

## Data Availability

Data availability is not applicable.
