# Supplementary material for: Racial disparity in the genomics of precision oncology of prostate cancer
Source: Cancer Rep (Hoboken). 2023 Aug 10;6(Suppl 1):e1867. doi: 10.1002/cnr2.1867 (PMC10440844; doi:10.1002/cnr2.1867)
Supplement: Supplementary file 1 — Supplemental Figure 1. Risk allele frequencies of 269 PCa SNPs, EAM versus AAM. The SNPs data were collected from Conti et al. study.19 [file CNR2-6-e1867-s001.docx]

**Supplemental Figure:**

Supplemental Figure 1. *Risk Allele Frequencies of 269 PCa SNPs, EAM Vs. AAM*The SNPs data were collected from Conti et al. study^19^.
